# Editorial: Extremophiles: tolerance mechanisms and use in crop improvement

**DOI:** 10.3389/fpls.2023.1233202

**Published:** 2023-06-20

**Authors:** Rengin Ozgur Uzilday, Showkat Ahmad Ganie

**Affiliations:** ^1^ Department of Biology, Faculty of Science, Ege University, Izmir, Türkiye; ^2^ Plant Molecular Science and Centre of Systems and Synthetic Biology, Department of Biological Sciences, Royal Holloway University of London, Egham, United Kingdom

**Keywords:** extremophiles, crop improvement, plant growth, stress tolerance, abiotic stress

Climate change increases both the intensity and frequency of environmental stresses such as temperature extremes, drought, and salinity. However, the majority of crop plants are sensitive to these stresses and huge yield penalties occur when they are exposed to these sub-optimal conditions. Crop plants cannot grow in natural environments where the abiotic stresses are extreme such as deserts, saline and high metal soils, Arctic and Antarctic regions, etc. In contrast, the extremophile plants or extremophytes, including halophytes (growing in soils with high salinity), xerophytes (growing in dry and arid environments), resurrection plants (survive almost complete dehydration or desiccation even over years), metal hyperaccumulators (growing in soils or waters with extreme levels of metals and concentrate these metals in their living tissues), etc. have evolved to complete their life cycles in these marginal habitats and have developed various mechanisms to cope with these stresses at anatomical, physiological, biochemical, and molecular levels. It is thus important to understand these mechanisms in extremophiles and to use that knowledge in the improvement of stress tolerance-related traits in cultivated crops to maximize the land area for the crop cultivation. In fact, in the past few decades, especially after the development of NGS technologies, plant scientists have shown a great interest in understanding the molecular genetic background of tolerance mechanisms in these astonishing plants and in transferring this knowledge to cultivated crops. This is especially feasible in the case of crop species which are closely related to an extremophile(s). Despite these efforts, the research in this area is in its infancy and the current knowledge is not enough, which warrants more studies on the extremophiles.

In this regard, this Research Topic aimed to collect studies related to deciphering stress tolerance mechanisms in extremophiles that have the potential to be used in the improvement of crop stress tolerance for safeguarding yield. Studies related to understanding novel tolerance mechanisms of extremophiles are of special importance since they form the fundamental knowledge required for crop improvement.

Halophytes evolved to survive under extreme saline conditions and some even require saline conditions for their normal metabolic function. Comparative studies between halophytes and glycophytes (grow in low sodium ecosystems and are sensitive to high salinity) are widely conducted in recent years to reveal how halophytes tolerate extreme conditions. Guo et al. used genomic and transcriptomic strategies to understand the regulation of gene expression during salinity in model halophyte *Puccinellia tenuiflora* (Poaceae) and compared its response with several glycophytic Poaceae plants. This study unveiled the evolutionary network of salinity tolerance genes in *P. tenuiflora* and found that potassium channel and transporter gene families of *P. tenuiflora* were positively selected during evolution which cause higher K content under salinity stress.

*Tamarix ramosissima* (Tamaricaceae) is naturally widespread in harsh environments including shelterbelt forests which are important to reduce desertification in arid regions. It is a perennial shrub and its survival strategy under harsh conditions in its habitat are unknown. In such areas, precipitation is very low which requires the evolution of unique water use strategies for plants to growth and survive. Liu et al. measured the stem sap flow (SSF) of *T. ramosissima* daily during growth season to understand the water use strategies and found that SSF highly depends on water availability. It was found that *T. ramosissima* mainly uses deep soil water and ground water under extreme water-limiting environment, and that photosynthetic active radiation, air temperature and vapor pressure deficit are the main environmental drivers for its SSF under these severe conditions.

Comparative studies between extremophiles and their stress sensitive crop relatives under stress conditions are also important ways to understand evolution of stress coping mechanisms. Plant genetic diversity for tolerance to harsh environments can also be evaluated through transposable element (TE) colonization. Wang et al. surveyed TE compositions of two mangrove *Sonnertia* species, viz. salt tolerant *S. alba* (Lythraceae) and less tolerant relative *S. caseolaris* (Lythraceae). By using different approaches such as phylogenic analysis of long terminal repeat retrotransposons, small RNA sequencing and whole-genome bisulfite sequencing, the authors demonstrated that *S. alba* shows richer TEs than that of *S. caseolaris*. Also, *S. alba* exceptionally showed a burst in *Gypsy* retrotransposons, but this burst had a weak effect on the expressions of salt stress genes, suggesting that expansion of *Gypsy* retrotransposons is pervasive in mangroves which, however, is not correlated with salt-responsive gene expression in *S. alba*. Importantly, the study suggests that although TEs could be pervasively induced by stress, their co-option for the regulation of host genes should be cautious.

Germination and early seedling growth are the key stages of plant life which are also sensitive to stress conditions. Nutrients are stored in cotyledons and resources required for establishment of photosynthetic machinery are provided by cotyledons in early development. Cao et al. studied a unique halophyte, *Suaeda aralocaspica* (Amaranthaceae), that forms enlarged cotyledons. Under extreme salinity, this species of extremophiles postpones the development of true leaves and stays with cotyledons nearly for two months to avoid harmful effects of saline conditions on plant growth. Cao et al. performed in-depth investigation of cotyledon morphology and metabolism to shed light on the intriguing adaptation mechanism of this species. Contents of some metabolites, including osmolytes, and the expression of stress-related genes were markedly high under salinity. Moreover, this study demonstrated the developmental strategy of *S. aralocaspica* to improve homeostasis in energy consumption for enhancing early seedling performance under extreme levels of salt stress.

Quinoa is a facultative halophyte with high nutritional value and is considered as a superfood that is attracting attention from consumers. Yan et al. studied the effects of salinity on the first steps of quinoa seed germination which is the imbibition stage. They used artificial NaCl and natural salt solutions for 8 h long imbibition and made transcriptomic and metabolomics analyses. Carbohydrate metabolism and antioxidant defense mechanisms were found to be regulating the seed initial imbibition during high salinity or under brackish water.

Preventing desertification is a main goal for combatting adverse effects of climate change, and maintaining the natural desert-tolerant vegetation for those arid areas is an ambitious motive. *Haloxylon aphyllum* (Amaranthaceae) is a xero-halophyte which is dominant species in deserts of Iran. Excess dust exposure of this species negatively affects its growth and performance. In the last decade, studies on the beneficial effects of plant growth promoting bacteria (PGPB) on plant performance under stress conditions have significantly increased. Zilaie et al. applied two halotolerant plant growth promoting rhizobacteria to halophyte *H. aphyllum* and studies its response to salinity and to stress caused by dust coverage. Application of beneficial bacteria decreased the reactive oxygen species (ROS) accumulation and enhanced antioxidant capacity which led to more effective growth under desert conditions. Results from this study suggest that these PGPB not only confer the stress tolerance to the stress sensitive plant species but can also improve the thriving ability of extremophiles.

Overall, the research studies highlighted in this editorial elucidate a few novel stress tolerance mechanisms in the extremophiles, which may have significant implications for plant biotechnology and crop improvement, especially for the development of stress-tolerant crop plants.

## Author contributions

All authors listed have made a substantial, direct, and intellectual contribution to the work and approved it for publication.

